# Integrative Analysis of *EPHX4* as a Novel Prognostic and Diagnostic Biomarker in Lung Adenocarcinoma

**DOI:** 10.3390/ijms26115095

**Published:** 2025-05-26

**Authors:** Pengze Liu, Yutong Chen

**Affiliations:** 1 Department of Pathology, School of Medicine, Jinan University, Guangzhou 510632, China; liupz5@mail2.sysu.edu.cn; 2 School of Medicine, Shenzhen Campus of Sun Yat-sen University, Sun Yat-sen University, Shenzhen 518107, China

**Keywords:** *EPHX4*, lung adenocarcinoma, prognostic biomarker, immune infiltration, diagnosis, cell cycle

## Abstract

Lung adenocarcinoma (LUAD) remains a leading cause of cancer-related mortality, necessitating the identification of novel biomarkers for improved prognosis and diagnosis. This study investigates the role of epoxide hydrolase 4 (*EPHX4*), a member of the epoxide hydrolase family, in LUAD. Using data sourced from The Cancer Genome Atlas (TCGA) and Genotype-Tissue Expression (GTEx) databases, which were subsequently validated by the Gene Expression Omnibus (GEO), we analyzed levels of *EPHX4* expression, mutation, and methylation in tumors versus normal tissues. Our findings revealed a significant upregulation of *EPHX4* in LUAD tissues compared to normal lung tissues (*p* < 0.001), correlating with poorer overall survival (OS), disease-specific survival (DSS), and progression-free interval (PFI). Furthermore, *EPHX4* exhibited considerable diagnostic potential, as demonstrated by an area under the curve (AUC) of 0.854 in a Receiver Operating Characteristic (ROC) analysis. Notably, *EPHX4* expression was associated with immune infiltration, specifically Th2 cells, neutrophils, and macrophages, along with immune checkpoint molecules including PD-L1, PD-L2, and TIM-3. Additionally, *EPHX4* was involved in pivotal tumor-associated pathways, particularly cell cycle regulation. In conclusion, an elevated *EPHX4* expression is indicative of poorer prognosis in LUAD and may play a role in immune evasion and cell cycle dysregulation, highlighting its potential as a promising biomarker for the diagnosis and prognostic prediction of LUAD.

## 1. Introduction

Lung cancer constitutes a significant and pressing worldwide health concern, as it stands as the primary cause of cancer-related deaths among individuals aged over 50, affecting countless lives and families across the globe. Non-small cell lung cancer encompasses 80–85% of all lung cancer diagnoses, with lung adenocarcinoma (LUAD) representing the most common histological category, making up roughly 40% of NSCLC instances [1]. The progression of LUAD is linked to genetic alterations, highlighting the importance of molecular analysis [2]. Although there have been improvements in therapeutic approaches, the five-year survival rate for LUAD continues to be low, typically between 18 and 20%, and is closely associated with the stage at which the disease is identified, underscoring the need for enhanced early detection techniques [3]. Current diagnostic methods rely primarily on imaging and biopsies, which are limited in their capacity to facilitate early diagnosis [4]. Consequently, the discovery of additional biomarkers and potential therapeutic targets remains essential for improving LUAD diagnosis, management, and prognostic outcomes.

Oxidative stress is fundamentally implicated in the initiation and progression of tumors [5]. Epoxide hydrolases (EHs), a modest group initially regarded as detoxifying enzymes, exhibit a significant connection to oxidative stress [6]. These enzymes catalyze the hydrolysis of epoxides by introducing water molecules, resulting in the formation of the corresponding vicinal diols [7]. Excessive stimulation of epoxy compounds may lead to mutagenic, toxic, and carcinogenic effects [8]. The EHs that have been most extensively researched include *EPHX1*, *EPHX2*, and *EPHX3* [9]. Among them, *EPHX1* and *EPHX2* stand out as the most significant members, with their altered expressions reported in several human cancers [9]. EPHX3 has been identified as a modulator of tumorigenesis across 13 different cancer types [10]. The findings suggest a strong correlation between members of the EPHX family and cancer development.

EPHX4 demonstrates substantial similarity to EPHX3 [10]. Nonetheless, limited research has explored the expression and clinical significance of *EPHX4* in cancer. Two recent investigations have indicated a possible link between *EPHX4* and the onset and progression of laryngeal squamous cell carcinoma and colorectal cancer [11,12]. Accordingly, the potential research value of *EPHX4* in lung adenocarcinoma, and potentially other cancers, warrants further investigation.

This research utilized an extensive methodology to examine the function of EPHX4 in lung adenocarcinoma (LUAD) by drawing on information from The Cancer Genome Atlas (TCGA) and the Gene Expression Omnibus (GEO) repositories. A thorough evaluation was conducted to determine the significance of *EPHX4* in diagnosis and prognosis, aiming to clarify the connection between its expression patterns and clinical outcomes. In order to deepen our comprehension, we carried out correlation analyses involving clinical variables and developed prognostic nomograms for estimating survival probabilities. Furthermore, we examined immune cell infiltration patterns and predicted immunotherapy response to assess the potential immunological implications of *EPHX4* expression. The research further encompassed an examination of genetic mutations and methylation profiles to investigate the genomic context linked to *EPHX4*. A differential expression assessment in high versus low *EPHX4* expression groups was performed to detect notable gene alterations, which was succeeded by functional enrichment evaluations to clarify the relevant biological mechanisms. More precisely, the investigation centered on the possible connection between *EPHX4* and cell cycle regulation. Ultimately, we developed a protein–protein interaction network to illustrate the connections between EPHX4 and other associated proteins. In summary, our results suggest that *EPHX4* may serve as a new prognostic and diagnostic biomarker for LUAD.

## 2. Results

### 2.1. EPHX4 Emerged as the Key Oxidative Stress-Related DEG in LUAD

**Figure 1:**
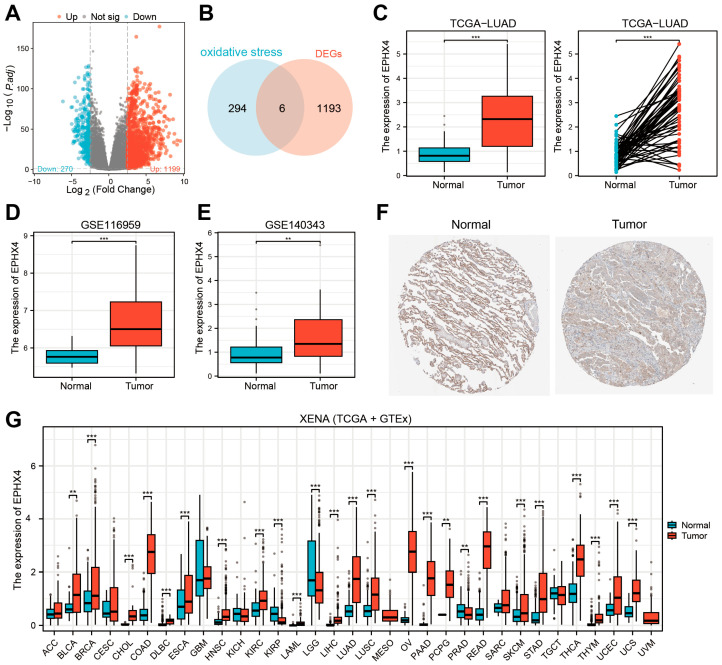
Upregulation of *EPHX4* in lung adenocarcinoma (LUAD) (**A**) Differential expression genes (DEGs) in the TCGA-LUAD dataset between tumors and normal tissues. (**B**) Intersection between DEGs and oxidative stress-related genes. (**C**) Comparison of *EPHX4* expression differences between tumors and normal tissues in unpaired and paired samples from the TCGA-LUAD dataset. (**D**,**E**) Validation of *EPHX4* expression difference in datasets GSE116959 and GSE140343. (**F**) Comparison of *EPHX4* protein levels in the HPA database (antibody HPA035067, 10×). (**G**) Detection of *EPHX4* expression in a pan-cancer dataset combining TCGA and GTEx. ** *p* < 0.01, *** *p* < 0.001.

An analysis of the TCGA-LUAD dataset demonstrated that, when compared to normal lung tissues, a total of 1199 genes were notably upregulated, while 270 genes showed significant downregulation in the tumor samples (Figure 1A). The intersection between the 1199 upregulated differentially expressed genes (DEGs) and genes linked to oxidative stress identified six specific genes (Figure 1B). The Receiver Operating Characteristic (ROC) analysis revealed that *EPHX4* possessed the highest potential for diagnostic accuracy (Figure S1). As a result, our subsequent analysis concentrated on *EPHX4*. As previously indicated, the expression of *EPHX4* was markedly increased (*p* < 0.001), and a further comparison of 58 paired LUADs and adjacent normal tissues reaffirmed these findings (Figure 1C). Consistent results were also obtained in the GSE116959 and GSE140343 cohorts (Figure 1D,E). Immunohistochemical (IHC) assessments of LUAD and normal lung tissues, derived from the Human Protein Atlas (HPA) database, validated the expression of *EPHX4* at the protein level (Figure 1F). Furthermore, a pan-cancer analysis that combined data from the TCGA and GTEx databases indicated significant overexpression of *EPHX4* in 22 different tumor types, including LUAD, alongside notable underexpression in 2 tumor types; however, variations in other tumor types did not achieve statistical significance (Figure 1G). Additionally, a single-cell analysis from four diverse sample sources within the TISCH database illustrated the expression of *EPHX4* in particular cell populations (Figure S2).

### 2.2. Elevated EPHX4 Expression Is Associated with Poor Prognosis in LUAD

**Figure 2:**
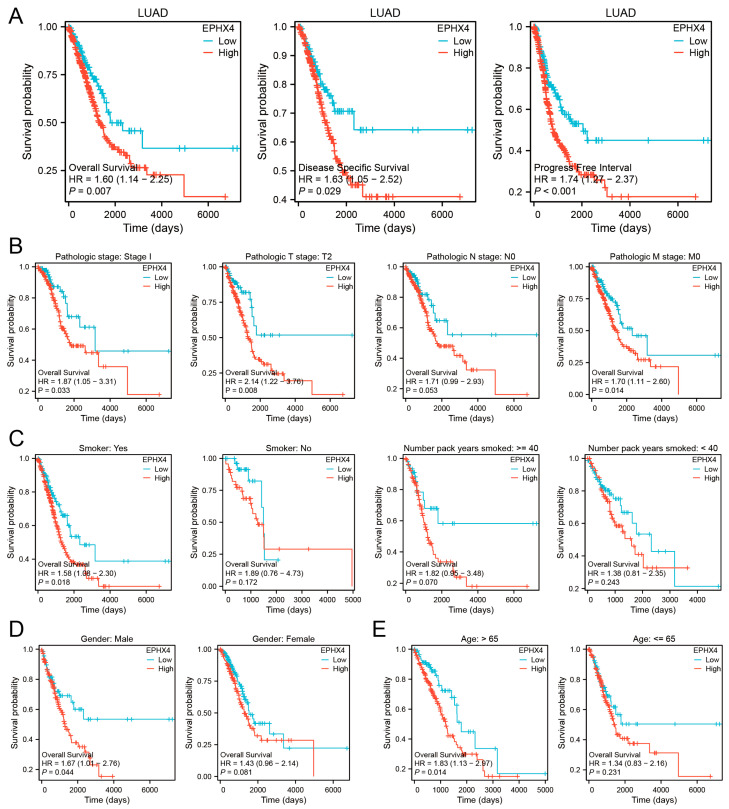
Prognostic value of *EPHX4* in overall LUAD patients and subgroups (**A**) OS/DSS/PFI survival curves for the TCGA-LUAD cohort. (**B**–**E**) OS comparisons between high and low *EPHX4* expression groups, with subgroup analyses based on tumor stage, smoking status, duration of smoking, gender, and age.

To evaluate the prognostic relevance of *EPHX4* in LUAD, a Kaplan–Meier survival analysis was conducted to investigate patient survival outcomes in relation to different levels of *EPHX4* expression. The analysis of the TCGA dataset revealed a significant association between high *EPHX4* expression and poor overall survival (OS) (HR = 1.60, *p* = 0.007), disease-specific survival (DSS) (HR = 1.63, *p* = 0.029), and progression-free interval (PFI) (HR = 1.74, *p* < 0.001) among LUAD patients (Figure 2A). Following the identification of high *EPHX4* expression as a potential risk factor for LUAD, we conducted further subgroup analyses to identify populations with enhanced predictive value. Utilizing a threshold of *p* < 0.05, *EPHX4* demonstrated significant predictive capability for prognosis in early-stage LUAD (Figure 2B). Furthermore, *EPHX4*’s predictive accuracy was notably greater in smokers compared to non-smokers, particularly among individuals with a smoking history exceeding 40 pack years (Figure 2C). Moreover, the predictive significance of *EPHX4* was more pronounced in male patients (Figure 2D) and those aged over 65 years (Figure 2E). Additionally, we executed Cox proportional hazards regression to assess OS, DSS, and PFI in patients from the TCGA-LUAD cohort. From these analyses, we constructed nomograms to estimate survival probabilities (Figure S3).

### 2.3. EPHX4 Is a Potential Diagnostic Biomarker for LUAD

**Figure 3:**
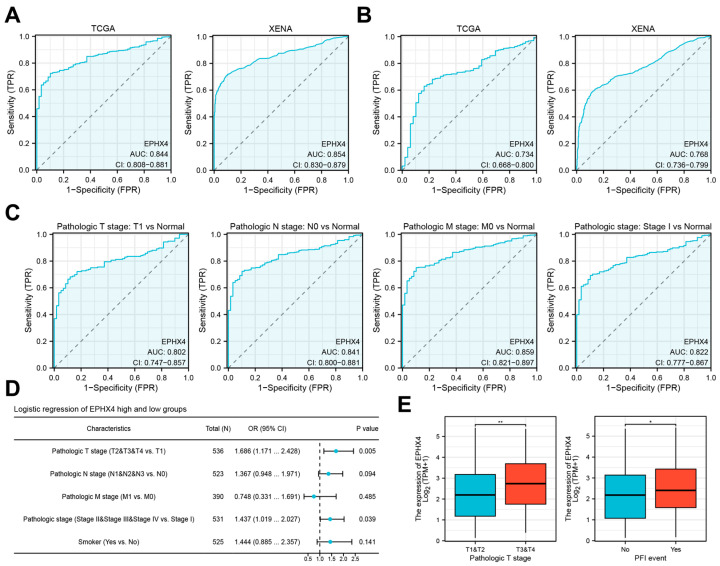
Diagnostic value and clinical significance of *EPHX4* (**A**,**B**). The ROC analysis shows the diagnostic value of *EPHX4* in differentiating between LUAD and lung squamous cell carcinoma (LUSC). (**C**) Diagnostic ROC curve for *EPHX4* in early LUAD subgroup. (**D**) Logistic regression analysis results of *EPHX4* expression with clinical variables in LUAD. (**E**) Differences in *EPHX4* expression across different tumor pathological stages and survival periods in LUAD. * *p* < 0.05, ** *p* < 0.01.

A ROC analysis indicated that *EPHX4* possesses considerable discriminative capability in the clinical identification of both LUAD (Figure 3A) and lung squamous cell carcinoma (LUSC) (Figure 3B), with expression levels proficiently differentiating tumor tissues from their normal counterparts. The area under the curve (AUC) signified its exceptional diagnostic efficacy in LUAD. Further investigations were concentrated specifically on LUAD. The ROC subgroup analysis revealed that *EPHX4* exhibited notable discriminative value for LUAD patients categorized under pathological stages T1/N0/M0 and stage I, highlighting its potential as a biomarker for the early detection of LUAD (Figure 3C). The univariate logistic regression analysis uncovered a significant correlation between *EPHX4* expression levels and both the pathological T stage and the pathological N stage (*p* < 0.05) (Figure 3D). Notably, LUAD patients classified under the T3 and T4 stages displayed heightened *EPHX4* expression when compared to those in the T1 and T2 stages. Furthermore, individuals who experienced recurrence or metastasis manifested elevated levels of *EPHX4* expression (Figure 3E).

### 2.4. EPHX4 Is Significantly Linked to Immune Cell Infiltration

**Figure 4:**
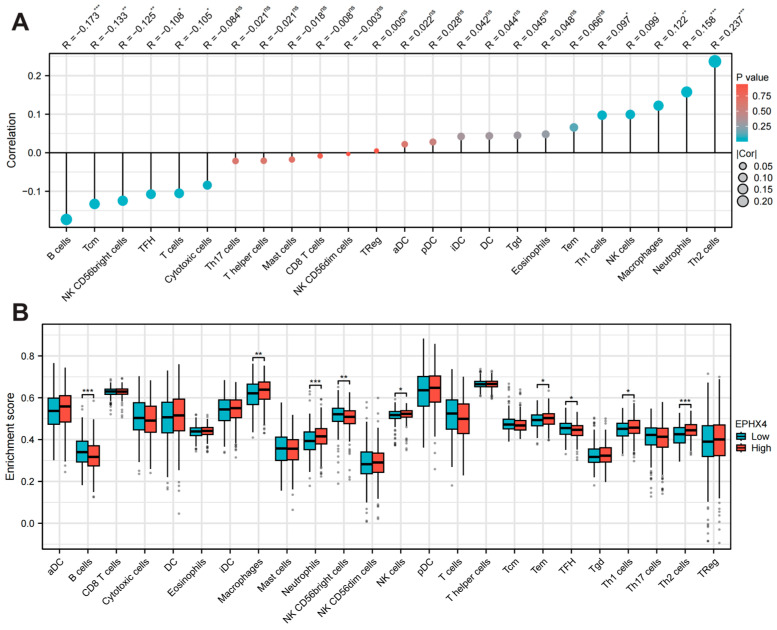
Immune infiltration analysis of *EPHX4*. (**A**) Correlation analysis of *EPHX4* expression with immune cell infiltration levels. (**B**) Comparison of various immune cell infiltration levels between high and low *EPHX4* expression groups. * *p* < 0.05, ** *p* < 0.01, *** *p* < 0.001.

Employing the ssGSEA methodology, we assessed the association between the expression levels of *EPHX4* and the infiltration of 24 unique immune cell types. Our results demonstrated a positive correlation between *EPHX4* expression and the presence of Th2 cells, neutrophils, and macrophages, while a negative correlation was noted with B cell infiltration (Figure 4A). Following this, we stratified the LUAD samples into categories based on high and low *EPHX4* expression, which revealed differences in immune cell enrichment scores. In the cohort exhibiting elevated *EPHX4* expression, we observed substantial increases in the populations of Th2 cells, neutrophils, macrophages, natural killer (NK) cells, effector memory T (Tem) cells, and Th1 cells (all *p* < 0.05). Conversely, we identified a significant reduction in B cells, follicular helper T (Tfh) cells, and NK CD56^+^ cells (all *p* < 0.05) (Figure 4B). Moreover, data obtained from the TIMER2.0 database reinforced a significant positive correlation between *EPHX4* expression and the infiltration of CD4+ T cells, neutrophils, and macrophages, alongside a notable negative correlation with B cell infiltration (Figure S4), thus validating the outcomes from the preceding ssGSEA analysis.

### 2.5. EPHX4 Is Associated with Immune Checkpoints and Immunotherapy Response

**Figure 5:**
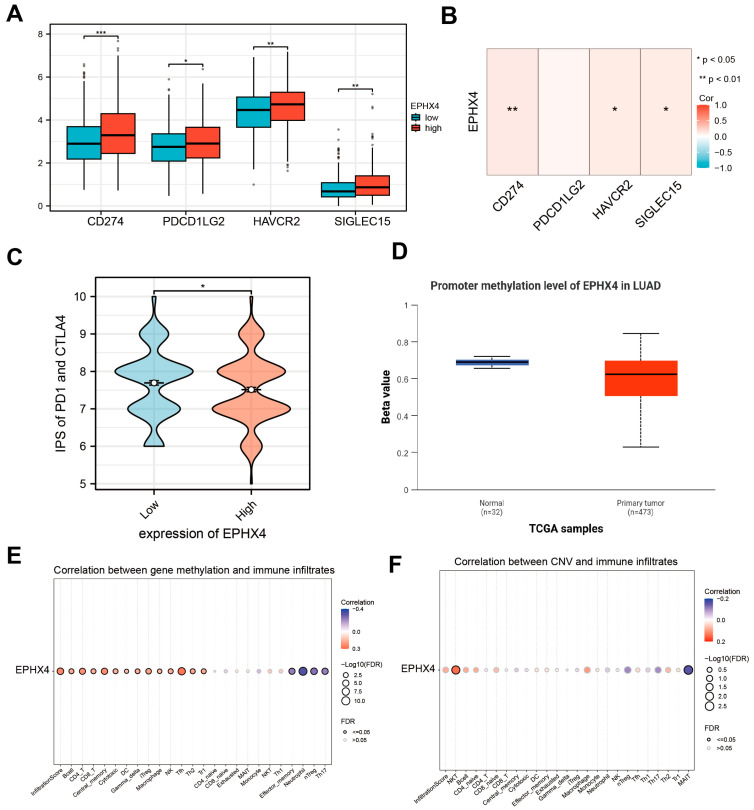
Correlation between *EPHX4* and immune checkpoints as well as immunotherapy. (**A**) The levels of immune checkpoint genes in the high versus low *EPHX4* expression groups. (**B**) Correlation of *EPHX4* expression with immune checkpoint genes. (**C**) Difference in IPS score between the high versus low *EPHX4* expression groups. (**D**) Comparison of *EPHX4* methylation levels. (**E**,**F**) Correlation of *EPHX4* methylation and CNV with immune infiltration. * *p* < 0.05, ** *p* < 0.01, *** *p* < 0.001.

We conducted a detailed exploration of the potential correlation between *EPHX4* and immune checkpoints, as well as the response to immunotherapy. Our findings demonstrated a notable increase in the transcriptional levels of four specific genes within the *EPHX4* high-expression subset: CD274, which encodes for PD-L1; PDCD1LG2, responsible for PD-L2; HAVCR2, encoding TIM-3; and SIGLEC15, which encodes Siglec-15 (Figure 5A). Furthermore, the correlation heatmap depicted a strong positive relationship between *EPHX4* expression and the levels of CD274, HAVCR2, and SIGLEC15 (Figure 5B). Additionally, an analysis utilizing the TCIA database revealed that the Immune Profiling Score (IPS) was significantly lower in the *EPHX4* high-expression cohort, indicating a reduced efficacy of immunotherapy (Figure 5C). Moreover, data from the UALCAN database suggested that the methylation levels of the *EPHX4* promoter were decreased in tumor tissues (Figure 5D), a finding that was supported by the MethSurv database (Figure S5). Importantly, the GSCA database highlighted a significant relationship between the *EPHX4* promoter methylation and the infiltration levels of various immune cells in LUAD tissues, whereas the association between the *EPHX4* copy number variations (CNVs) and the immune infiltration largely lacked statistical significance (Figure 5E,F).

### 2.6. Relationship Between EPHX4 Expression and Somatic Variants

**Figure 6:**
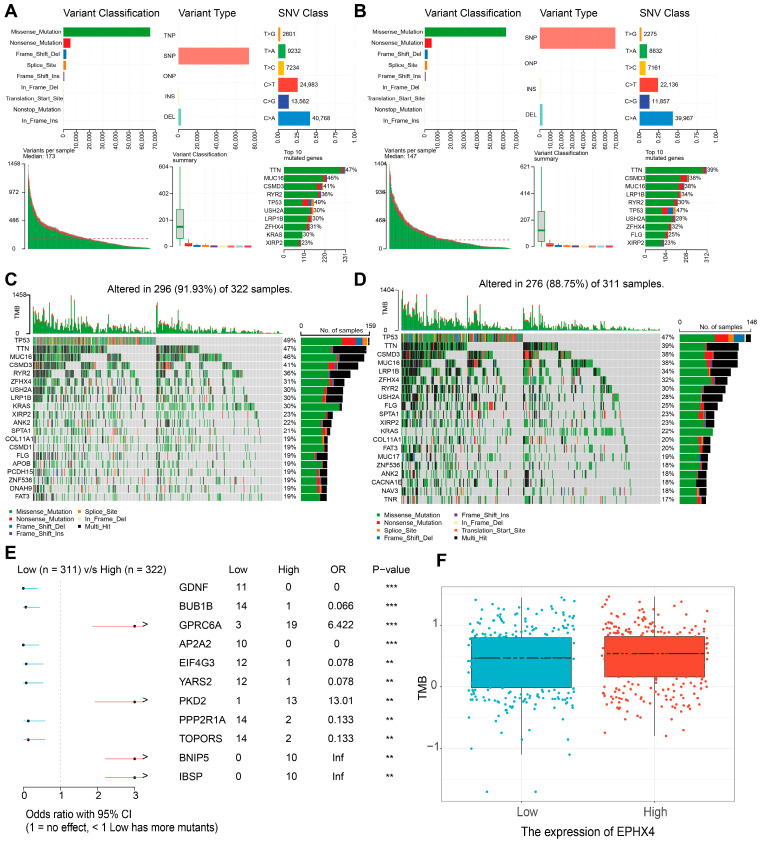
Relationship between somatic mutations and *EPHX4* expression. (**A**,**B**) Cohort summary plots of the high and low *EPHX4* expression groups, displaying the distribution of variants according to variant classification, type, and SNV class. (**C**,**D**) Oncoplots of the mutated genes in the high and low *EPHX4* expression groups. (**E**) Forest plot displaying differentially mutated genes between the high and low *EPHX4* expression groups. (**F**) Box-and-dot plot showing differences in tumor mutation burden (TMB) between the high and low *EPHX4* expression groups.** *p* < 0.01, *** *p* < 0.001.

In the cohort characterized by the elevated *EPHX4* expression, the five genes exhibiting the highest mutation frequencies were identified as TTN (47%), MUC16 (46%), CSMD3 (41%), RYR2 (36%), and TP53 (49%). Conversely, in the group with reduced *EPHX4* expression, the most frequently mutated genes included TTN (39%), CSMD3 (38%), MUC16 (38%), LRP1B (34%), and RYR2 (30%) (Figure 6A,B). The oncoprint visualizations highlighted variances among the top 20 genes with the highest mutation rates between the two expression groups, with the statistical significance noted (*p* < 0.05) (Figure 6C,D). Additionally, our analysis uncovered substantial differences in mutation counts between the high and low *EPHX4* expression cohorts (Figure 6E). Ultimately, the tumor mutational burden (TMB) was elevated in the group exhibiting high *EPHX4* expression compared to their low-expression counterparts (Figure 6F). Furthermore, through the application of cBioPortal, we identified genetic alterations in *EPHX4*, which demonstrated a positive correlation between the *EPHX4* copy number variations (CNV) and the mRNA expression levels (Figure S5).

### 2.7. EPHX4 Might Be Significantly Involved in the Regulation of the Cell Cycle

**Figure 7:**
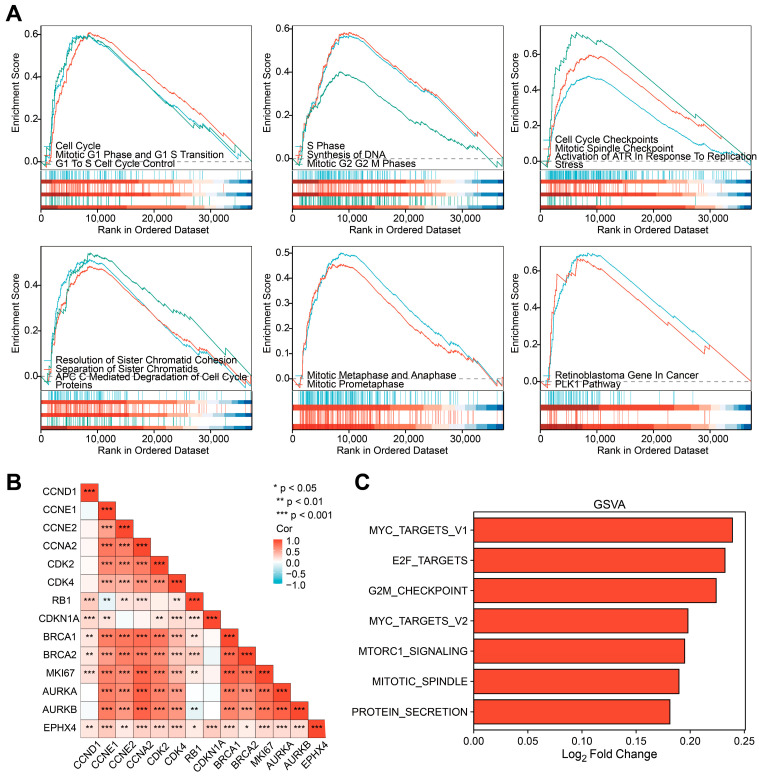
GSEA and GSVA functional analysis of *EPHX4*. (**A**) GSEA gene set enrichment results. (**B**) Correlation between *EPHX4* and cell cycle-related genes. (**C**) GSVA gene set enrichment results.

We further explored the underlying association between EPHX4 and diverse biological processes by conducting a GSEA analysis. The findings revealed that the *EPHX4* expression was significantly correlated with the entire cell cycle process (Figure 7A). Specifically, the *EPHX4*-related pathways encompassed core cell cycle stages, checkpoints, key regulatory steps, and regulatory molecules. These pathways involved the transition from the G1 phase to the S phase, the S phase (DNA replication), and the transition from the G2 phase to the M phase, as well as the core stages of mitosis: prophase, metaphase, and anaphase. They also included the regulation of checkpoints for DNA damage and replication errors, such as the role of ataxia telangiectasia and Rad3-related protein kinase (ATR) during replication stress. Key regulatory steps involved spindle formation, sister chromatid separation, and cyclin degradation mediated by the anaphase-promoting complex/cyclosome (APC/C). Additionally, regulatory molecules such as the retinoblastoma protein (Rb) and polo-like kinase1 (PLK1) were implicated. Subsequently, we focused on analyzing the co-expression of *EPHX4* with genes encoding cell cycle-related proteins, including those encoding cyclins CCND1, CCNE1, CCNE2, CCNA2, cyclin-dependent kinases CDK2 and CDK4, cell cycle checkpoint protein CDKN1A, DNA repair-related proteins BRCA1 and BRCA2, Ki-67, and Aurora kinases AURKA and AURKB. The correlation heatmap showed that these genes were significantly correlated in TCGA-LUAD and significantly associated with *EPHX4* expression (Figure 7B). Consistently, the co-expression heatmap indicated that these genes significantly co-expressed with *EPHX4* in TCGA-LUAD (Figure S6). The results of the GSVA analysis further displayed the relationship between *EPHX4* and the cell cycle. In contrast to the *EPHX4* low-expression group, essential cell cycle pathways, including MYC targets, E2F targets, G2/M checkpoints, and mitotic spindle, were significantly upregulated in the *EPHX4* high-expression group (Figure 7C).

Subsequently, after conducting the GSEA and GSVA analyses, we undertook GO and KEGG functional evaluations, with the objective of clarifying the potential biological roles attributed to EPHX4 in different ways. Between the high and low *EPHX4* expression groups, a total of 1157 protein-coding genes exhibited differential expression. Further functional enrichment analysis of the 25 significantly differentially expressed genes (*LYZL2*, *GSTA2*, *EDDM3B*, *SLC17A2*, *SLC5A5*, *CT45A1*, *PGC*, *ALB*, *SCGB3A1*, *LHX1, CGB8*, *ONECUT3*, *PSG1*, *PRSS2*, *SPRR2F*, *SPRR2E*, *EPHX4*, *SLC6A15*, *CGB5*, *SPRR2D, ARHGAP36*, *NTSR1*, *C6orf15,* and *PRSS1*) was conducted subsequently. Results indicated that the main biological processes (BPs) involved multi-organism reproductive processes, epoxygenase P450 pathway, epidermal cell differentiation, and negative regulation of hydrolase activity. Key molecular functions (MFs) involve activities of endopeptidase inhibitors, endopeptidase regulators, and signaling receptor activators, as well as growth factor receptor binding. The KEGG analysis showed chemical carcinogenesis-DNA adducts pathway as a result (Figure S6).

### 2.8. EPHX4-Related Proteins and Their Functional Enrichment Analyses

**Figure 8:**
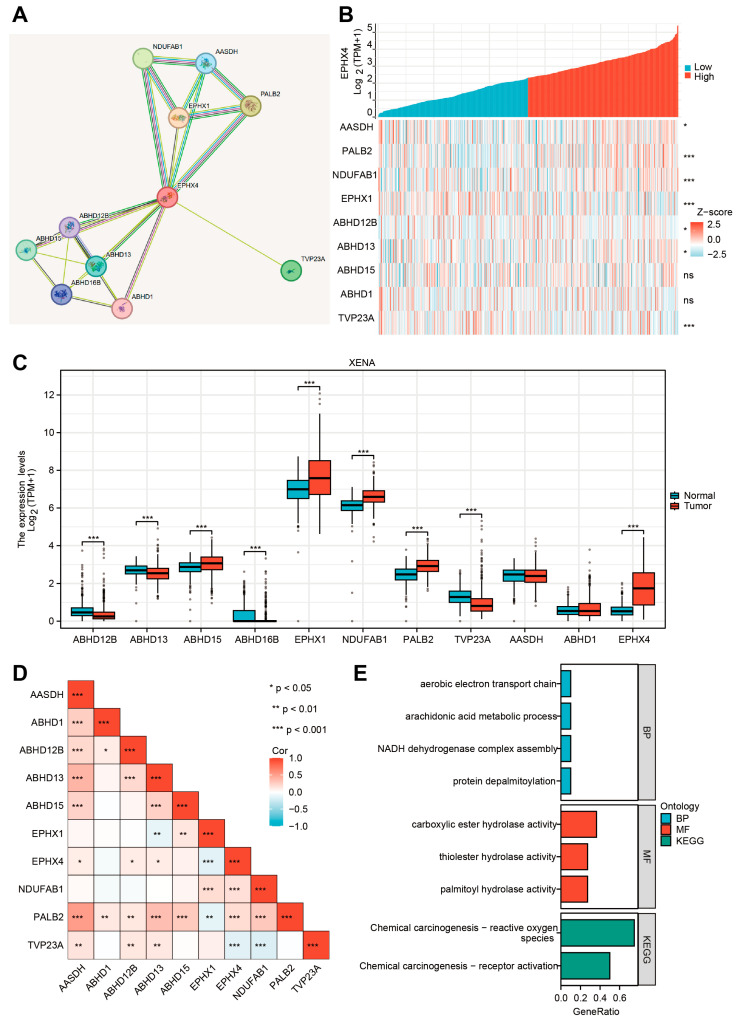
EPHX4-related proteins and their functional analysis. (**A**) PPI network of EPHX4-related proteins. (**B**) Co-expression heatmap of *EPHX4* with related genes. (**C**) Expression of *EPHX4*-related genes in LUAD. (**D**) Correlation between *EPHX4* and related genes. (**E**) GO/KEGG functional enrichment analysis of *EPHX4*-related genes. ns: *p* ≥ 0.05; * *p* < 0.05; ** *p* < 0.01; *** *p* < 0.001.

Functioning as a component of the broad protein network, EPHX4 engages in coordinated interactions with other proteins. In order to offer a broader perspective besides our previous analysis, we used the STRING database to further look into EPHX4-related proteins. Utilizing the STRING database, we constructed a protein–protein interaction network of proteins related to EPHX4. This analysis identified AASDH, PALB2, NDUFAB1, EPHX1, ABHD12B, ABHD13, ABHD15, ABHD16B, ABHD1, and TVP23A as the proteins most closely associated with EPHX4 (Figure 8A). Genes encoding those proteins also exhibited co-expression with *EPHX4*, particularly *NDUFAB1*, *PALB2*, *TVP23A*, and *EPHX1* (Figure 8B), which were named as “*EPHX4*-related genes”. A comparative evaluation utilizing the UCSC Xena (TCGA-GTEx) database revealed that all *EPHX4*-related genes, apart from *AASDH* and *ABHD1*, demonstrated substantial expression variations of LUAD when contrasted with normal tissues (*p* < 0.001) (Figure 8C). As seen in Figure 8D, in TCGA-LUAD, *EPHX4* showed a strong significant correlation with *NDUFAB1*, *PALB2*, and *TVP23A*, which were also up or downregulated in tumors as compared to normal tissues (Figure 8C). Interestingly, PALB2 has a close interaction with *BRCA1* and *BRCA2*, which could lead to malignant tumors [13]. *NDUFAB1* encodes an important subunit of NADH (Nicotinamide adenine dinucleotide) and plays a key role in cellular respiration [14]. TVP23A plays a role in maintaining the function of the Golgi apparatus [15]. Future research could pay attention to the underlying interaction between *EPHX4* and those genes. Finally, a functional enrichment analysis showed that these *EPHX4*-related genes derived from the STRING database are involved in the aerobic electron transport chain, the arachidonic acid metabolic process, the NADH dehydrogenase complex assembly, and protein depalmitoylation. Additionally, these genes exhibited enrichment for activities of carboxylic ester hydrolase, thioester hydrolase, and palmitoyl hydrolase, as well as participation in chemical carcinogenesis, including “reactive oxygen species” and “receptor activation” pathways (Figure 8E).

## 3. Discussion

Lung cancer is the most common type, with roughly 2.5 million newly diagnosed cases, posing a significant challenge to global health. In recent years, targeted therapy has yielded positive outcomes in lung cancer patients. However, its efficacy in preventing tumor progression is still limited [16]. Notwithstanding progress in innovative therapeutic approaches, the survival rate continues to be unsatisfactory [16]. The limited criteria for personalized therapies, along with the emergence of drug resistance, hinder some patients from gaining therapeutic benefits [17]. Traditional tumor indicators, including cancer antigen 125 and carcinoembryonic antigen, are commonly utilized. Nonetheless, their effectiveness is compromised by inadequate sensitivity and specificity, largely attributable to the prevalence of non-malignant conditions [17]. As a result, identifying new molecular indicators and treatment targets is essential for enhancing both the diagnosis and the therapeutic results in LUAD.

This research pinpointed *EPHX4* as a possible biomarker for LUAD, as our findings demonstrated a notable increase in *EPHX4* expression in neoplastic tissues relative to the surrounding normal tissues. Our findings demonstrate that high *EPHX4* expression correlates with poorer OS, DSS, and PFI in LUAD patients. Furthermore, ROC analyses indicate that *EPHX4* holds promise as a diagnostic tool, particularly in early-stage LUAD. We utilized a range of analytical techniques, such as evaluations of immune infiltration, co-expression studies of immune checkpoint genes, and the IPS derived from the TCIA database. These collectively reinforce the notion that *EPHX4* serves not only as a prognostic indicator, but may also be instrumental in the TME as well as immune response. In the context of somatic mutation analysis, our study revealed a correlation between *EPHX4* expression and an increased somatic mutation load. Furthermore, we identified a higher prevalence of mutations in the TTN and MUC16 genes within the cohort exhibiting elevated *EPHX4* expression compared to those with reduced *EPHX4* expression. An analysis of methylation patterns demonstrated that *EPHX4* exhibited diminished methylation levels in lung adenocarcinoma (LUAD) tissues when contrasted with normal lung tissues. Utilizing the STRING database, we constructed a PPI network that included proteins related to EPHX4. Last but not least, the results we gathered imply that EPHX4 plays a crucial and significant role in the intricate regulation of the cell cycle, influencing various phases and processes that are essential for proper cellular function and division. In conclusion, our findings advocate for the integration of *EPHX4* into clinical practices as a promising strategy to improve the diagnosis and treatment of LUAD.

The nature of the interactions between tumor cells and immune cells present in the TME influences the efficacy of anti-tumor responses [18]. We demonstrated notably positive correlations between the expression of *EPHX4* and different immune cell populations, especially Th2 cells, neutrophils, and macrophages. A specific subgroup of CD4+ T helper cells, known as Th2 cells, are essential in regulating immune responses, especially in B-cell-mediated humoral immunity. Emerging evidence suggests that within certain tumor microenvironments, Th2-dominated immune responses may contribute to tumor progression by shaping an immunosuppressive milieu and secreting cytokines, which can inhibit effective anti-tumor immunity and, in some contexts, promote tumor growth [18]. Our result suggests that EPHX4 may contribute to a tumor-promoting immune environment, potentially serving as a therapeutic target to modulate Th2 responses in LUAD. Neutrophils, which play a key role in innate immunity, have also been linked to the progression of cancer. Emerging research indicates that neutrophils associated with tumors can display characteristics that promote tumor growth, such as cancerous metabolic reprogramming and inhibiting adaptive immune responses [19]. The strong positive association between *EPHX4* expression and neutrophil infiltration in LUAD suggests that EPHX4 might play a role in the recruitment or activation of neutrophils within the tumor microenvironment, thereby reinforcing the proposition that EPHX4 may serve as a critical factor in determining the immune profile of LUAD. Tumor-associated macrophages (TAMs) constitute essential elements of the tumor microenvironment (TME). Recent studies have shown that tissue-resident macrophages can be induced to transition into SPP1⁺ TAMs, which in turn promote tumorigenesis and may potentially contribute to resistance to immunotherapy. [20]. The positive association between *EPHX4* expression and macrophage infiltration suggests that EPHX4 may modulate macrophage towards a phenotype that supports tumor growth and immune evasion [18]. Further exploration of *EPHX4* may provide potential strategies aimed at reprogramming TAMs to enhance anti-tumor immunity in LUAD.

Cancer immunotherapy aims to elicit a cellular immune response, particularly one facilitated by T cells that can specifically target and destroy tumors. Immune checkpoints, which are proteins produced by certain immune cells and cancer cells, play a crucial role in determining the effectiveness of immunotherapy. Recent studies have identified several immune checkpoints associated with LUAD, including TIM-3, CD200, and PD-1 [16]. Nevertheless, the impact of immunotherapy is hindered by the absence of reliable biomarkers for identifying potential therapeutic responders in LUAD, leading to many patients not deriving benefits from such treatments [16]. *EPHX4* shows significant co-expression with CD274, HAVCR2, and SIGLEC15, indicating a positive correlation that suggests its potential influence on immunotherapy through immune checkpoint modulation. CD274 encodes programmed death-ligand 1 (PD-L1), the principal ligand for PD-1, which is widely expressed in both tumor and immune cells and inhibits T cell activity via the PD-1/PD-L1 interaction [16]. Disrupting the PD-1/PD-L1 signaling pathway is a fundamental strategy in contemporary cancer immunotherapy, exemplified by agents such as pembrolizumab and atezolizumab. The HAVCR2 gene encodes TIM-3, an essential inhibitory receptor associated with T cell exhaustion, which is co-expressed with PD-1 in tumors and chronic infections, leading to a notable reduction in T cell functionality [16]. Furthermore, SIGLEC15 encodes Siglec-15, which is predominantly expressed in macrophages, osteoclasts, and select tumor cells [21]. Siglec-15 has the potential to downregulate T cell function through mechanisms that do not involve MHC-I [21]. Thus, EPHX4 may enhance the expression of immune checkpoints, facilitating immune evasion in lung adenocarcinoma and contributing to its progression. Finally, the lower Immune Profile Score (IPS) observed in the high *EPHX4* expression group indicates that EPHX4 could impact tumor immune evasion mechanisms, potentially reducing responses to anti-CTLA-4 and anti-PD-1 therapies.

Functional enrichment analyses using GSEA and GSVA revealed that EPHX4 plays a role in several critical cell cycle processes, such as the progression from the G1 to S phase (involving the activation of Cyclin D-CDK4/6), DNA replication during the S phase, regulation of checkpoints for DNA damage and replication errors, as well as sister chromatid separation, among additional functions. This reveals a potential mechanism through which EPHX4 promotes cancer, offering insights and a basis for future research. In order to anticipate the possible molecules interacting with *EPHX4* during the cell cycle, we analyzed its co-expression patterns and associations with numerous genes related to the cell cycle. We found that *EPHX4* exhibited significant co-expression with key cell cycle genes, including *CCND1*, *CCNE1*, *CCNE2*, *CCNA2*, *CDK2*, *CDK4*, *CDKN1A*, *BRCA1*, *BRCA2*, *MKI67*, *AURKA*, and *AURKB*. CCND1 (Cyclin D1) is a core regulatory factor in the G1 phase. Through interaction with and stimulation of CDK4/6 kinases, it induces phosphorylation of the retinoblastoma protein (Rb), facilitating the progression from the G1 to S phase [22]. CCNE1/CCNE2 (Cyclin E1/E2) is briefly expressed in late G1, forming a complex with CDK2, further phosphorylating Rb and activating DNA replication-related genes (such as MCM and CDC6), acting as a “molecular switch” for entry into the S phase [23]. Cyclin A2 (CCNA2) serves a dual function in cellular regulation: it associates with CDK2 during the S phase to facilitate the onset of DNA replication and interacts with CDK1 during the G2/M phase to control centrosome maturation and chromatin condensation, thereby acting as a “bistable regulator” within the cell cycle [23]. CDK4 and CDK2, as core kinases, coordinate phase transitions by phosphorylating different substrates (such as Rb, p27, and CDC25). Inhibitors of CDK4, including abemaciclib, have been employed in the management of breast cancer that is positive for hormone receptors [24]. The gene CDKN1A (p21), which is under the control of p53, prevents the progression from the G1 to S phase or from the G2 to M phase by suppressing Cyclin–CDK complexes, particularly those involving Cyclin E/A-CDK2, thus establishing its significance in the DNA damage checkpoint [23]. BRCA1/BRCA2 form a replication stress response complex during the S phase, repairing double-strand breaks through homologous recombination (HR) and maintaining replication fork stability. BRCA1 is involved in G2/M checkpoint activation, while BRCA2 mutations lead to HR defects [25]. Ki-67, through its association with nucleolar chromatin, serves as a commonly utilized indicator of cell proliferation. Its expression level exhibits a strong correlation with tumor grading and prognosis [26]. The mitotic regulatory network comprises AURKA/AURKB (Aurora kinase A/B). AURKA plays a role in centrosome maturation and spindle formation, whereas AURKB guarantees the accurate segregation of sister chromatids through the phosphorylation of histone H3 at Ser10. When both are overexpressed, it leads to multipolar spindles and chromosome missegregation, which is closely associated with tumor aneuploidy [27]. Collectively, these genes exacerbate tumor aggressiveness by dysregulating cell cycle checkpoints, enabling replicative immortality, and remodeling the tumor microenvironment to facilitate dissemination. *EPHX4* exhibits substantial co-expression with the aforementioned genes, indicating its potential involvement in the modulation of cell cycle-associated biological processes, which may influence tumor onset and development. In the past, the functional significance of EPHX4 remained poorly defined, and forthcoming research could prioritize clarifying its precise role within the cell cycle.

In addition to GSEA and GSVA, GO/KEGG analyses indicated that DEGs were primarily involved in processes such as growth factor receptor binding, reproductive processes, chemical carcinogenesis-DNA adduct pathways, and metabolic pathways, which are critical for tumor growth and survival. This evidence further suggests that EPHX4 may play a pivotal and crucial role in promoting not only the intricate process of cell division, but also the complex and multifaceted phenomenon of tumorigenesis, thereby potentially influencing the development, progression, and aggressiveness of various types of cancers.

EPHX4′s functional role in tumor biology remains largely unexplored in previous literature. Given the paucity of prior reports, the results derived from this investigation delineate a novel avenue for future investigation, which may contribute to our comprehension of the molecular mechanisms associated with LUAD. Hopefully, our findings also lay the groundwork for subsequent studies focused on creating targeted treatments that may enhance patient outcomes through the modulation of EPHX4-related pathways.

This study presents significant findings regarding the role of *EPHX4* in LUAD. However, it is essential to acknowledge its limitations. One major constraint that impacts the reliability of the findings is the absence of wet-lab experiments designed to validate the computational predictions, a situation that may lead to considerable uncertainties regarding the biological relevance and significance of the observed associations that have been identified through computational methods. Moreover, the sample size, while substantial, may not fully represent the diverse population of LUAD patients, potentially limiting the generalizability of the results. Clinical validation is also necessary to confirm the prognostic and diagnostic value of *EPHX4* in a real-world setting, as the current analysis is primarily based on bioinformatics approaches.

In summary, *EPHX4* emerges as a significant player in LUAD, with implications for diagnosis, prognostic prediction, and the elucidation of underlying tumorigenesis mechanisms. Subsequent investigations can refer to our findings and explore *EPHX4* with further experimental studies to delineate the specific mechanisms by which *EPHX4* influences tumor biology and to evaluate its clinical utility, ultimately contributing to improved patient outcomes.

## 4. Materials and Methods

### 4.1. Flow Chart of the Present Study

**Figure 9:**
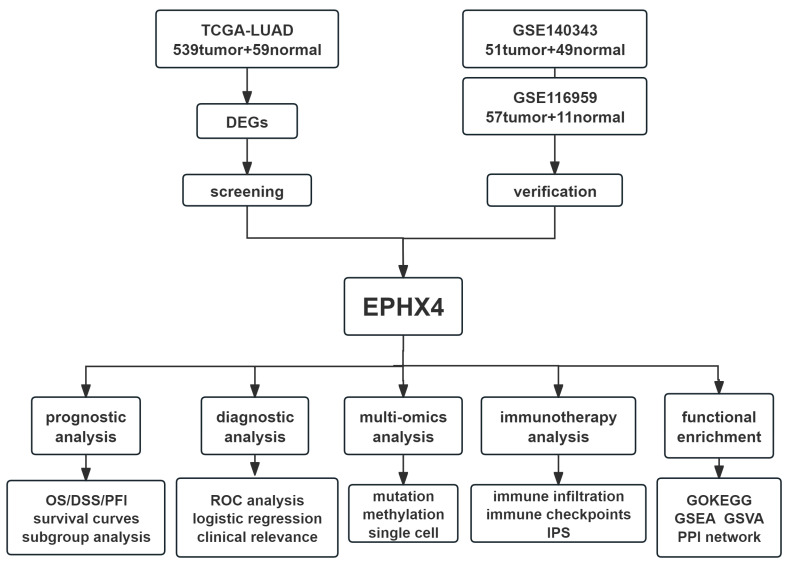
Flow chart of the present study.

A Flow chart was provided at the beginning to visually summarize the study’s framework and analytical process (Figure 9).

### 4.2. Data Acquisition and Differential Expression Analysis

The expression profiles of genes, along with relevant clinical data for 33 distinct tumor types, were obtained from The Cancer Genome Atlas (TCGA) and accessed via the UCSC Xena database (http://xena.ucsc.edu/). Furthermore, the UCSC Xena database provided integrated information on normal healthy tissues derived from the Genotype-Tissue Expression initiative (GTEx). “DESeq2” R package was utilized to standardize the raw counts matrix and identify DEGs in cancerous versus normal tissues [28]. We set the screening thresholds to |log_2_FoldChange| > 2.5 and *p*-adjusted below 0.05. DEGs were subsequently presented in a volcano plot. In addition, to ensure the reliability and verify the dependability, diverse expression data for LUAD were retrieved from the Gene Expression Omnibus (GEO), specifically utilizing datasets GSE116959 and GSE140343. To analyze *EPHX4* furtherly at the level of protein, immunohistochemical images of LUAD pathological samples alongside normal lung tissues were acquired from the Human Protein Atlas [29]. This investigation leveraged publicly available datasets, which negated the requirement for ethical approval and patient consent.

### 4.3. Screening of Candidate Genes and Diagnostic Value Analysis

The oxidative stress-related genes’ list is acquired from “GeneCards”, which can be accessed through https://www.genecards.org/. Following this, the upregulated differentially expressed genes were overlapped with genes associated with oxidative stress through the use of a Venn diagram. To identify a gene with optimal diagnostic potential in LUAD, ROC curves were generated for all genes within the intersection. Following a comparative analysis, *EPHX4* was selected as the candidate with the highest diagnostic value for further exploration. Subgroup ROC analyses of *EPHX4* were performed for different tumor pathological stages.

### 4.4. Prognostic Value Analysis

To thoroughly assess the intricate relationship between *EPHX4* expression levels and survival outcomes in patients diagnosed with LUAD, we meticulously analyzed several key metrics, including overall survival (OS), progression-free interval (PFI), and disease-specific survival (DSS) rates across distinct groups categorized by high and low levels of *EPHX4* expression. In addition to this primary analysis, we conducted further subgroup analyses that took into account a variety of clinical parameters, such as smoking status, the duration of smoking habits, age, and sex, as well as the pathological T, N, and M stages of the disease. The comprehensive analyses were executed with precision using the specialized “survival” and “survminer” R packages, which facilitated a robust examination of the data and allowed for a nuanced understanding of the factors influencing patient outcomes.

### 4.5. Correlation Analysis with Clinical Variables and Prognostic Nomogram Construction

The association between the expression levels of *EPHX4* and several clinicopathological variables—including age, sex, smoking history, and the T, N, and M stages of the disease—was investigated. A univariate Cox regression analysis was conducted utilizing the “survival” package in R to explore the relationship between *EPHX4* expression and clinical prognostic factors in individuals diagnosed with lung adenocarcinoma (LUAD). Furthermore, employing the “rms” package in R, prognostic nomograms were constructed.

### 4.6. Immune Cell Infiltration Analysis and Immunotherapy Outcome Prediction

Aiming to thoroughly investigate the underlying relationship between the expression levels of the *EPHX4* gene and the infiltration levels of 28 distinct immune cell types within the context of LUAD, we undertook a comprehensive single-sample gene set enrichment analysis (ssGSEA) by utilizing the “GSVA” R package [30,31]. Following this initial analysis, to ensure the reliability and to validate the dependability of our findings, we employed TIMER2.0 as a means of verification [32]. Furthermore, we sourced the immunophenotype score (IPS) data for the individuals within the TCGA-LUAD cohort from the Cancer Immunome Atlas, which can be accessed through https://tcia.at/. This information was further utilized for subsequent assessments to predict the effectiveness of immunotherapeutic interventions, including those involving CTLA-4 and PD-1 inhibitors.

### 4.7. Gene Mutation and Methylation Analysis

The mutation analysis data pertaining to LUAD were acquired through the utilization of the “TCGAbiolinks” R package. Subsequently, we conducted an analysis and visualization of the disparities in genomic variations between groups exhibiting high and low levels of *EPHX4* expression, employing the “maftools” R package. For every individual sample, the TMB was assessed and compared across groups categorized by high versus low *EPHX4* expression levels. Furthermore, the cBioPortal was utilized to explore mutations in *EPHX4* as well as copy number variations (CNVs), examining the association between alterations in *EPHX4* and mRNA expression levels [33]. Additionally, the UALCAN database was employed to investigate the methylation status of the *EPHX4* promoter in LUAD [34]. A correlation heatmap illustrating the interrelations among *EPHX4* methylation, CNVs, and immune cell infiltration in LUAD was derived from the Gene Set Cancer Analysis (GSCA), which can be accessed through the following network: https://guolab.wchscu.cn/GSCA/.

### 4.8. Functional Enrichment Analysis

The “clusterProfiler” and the “GSVA” R packages were employed for the purpose of functional annotation [35]. The gene set “c2.cp.all.v2022.1.Hs.symbols.gmt” was applied in this context. Statistical reliability was determined by utilizing false discovery rates that were less than 0.25. We explored interactions between proteins via the STRING database, leading to the development of an interaction network that includes EPHX4 and ten related proteins [36]. On the STRING website, a confidence interval cutoff of 0.4 was set as the minimum interaction score for network preparation, which suggests a medium interaction intensity or above.

### 4.9. Statistical Analysis

R (version 4.3.3) was employed in the statistical evaluations and visualization. To assess *EPHX4* expression, the Wilcoxon rank-sum test was applied to independent tissue samples, whereas matched samples were evaluated using a paired Student’s *t*-test. A *p*-value threshold of 0.05 was established as a rigorous criterion for determining statistical significance in all analyses conducted, ensuring that the results obtained would be deemed reliable and meaningful within the context of the research.

## 5. Conclusions

Elevated expression levels of *EPHX4* are associated with a poor prognosis in LUAD and may contribute to mechanisms of immune evasion and disruptions in the cell cycle. Consequently, *EPHX4* presents itself as a biomarker for both diagnosis and prognostic prediction in this patient population.

## Data Availability

Data is obtained from publicly available datasets, which is stated in the “Materials and Methods” section in detail.

## Supplementary Materials

The following supporting information can be downloaded at: https://www.mdpi.com/article/10.3390/ijms26115095/s1.

## References

[1] Adams S.J., Stone E., Baldwin D.R., Vliegenthart R., Lee P., Fintelmann F.J. (2023). Lung Cancer Screening. Lancet.

[2] Morel M., Long W. (2024). FBXL16 Promotes Cell Growth and Drug Resistance in Lung Adenocarcinomas with KRAS Mutation by Stabilizing IRS1 and Upregulating IRS1/AKT Signaling. Mol. Oncol..

[3] Wu J., Li L., Zhang H., Zhao Y., Zhang H., Wu S., Xu B. (2021). A Risk Model Developed Based on Tumor Microenvironment Predicts Overall Survival and Associates with Tumor Immunity of Patients with Lung Adenocarcinoma. Oncogene.

[4] Bae S., Yoon J.H., Moon H.J., Kim M.J., Kim E.-K. (2015). Breast Microcalcifications: Diagnostic Outcomes According to Image-Guided Biopsy Method. Korean J. Radiol..

[5] Hayes J.D., Dinkova-Kostova A.T., Tew K.D. (2020). Oxidative Stress in Cancer. Cancer Cell.

[6] Fretland A.J., Omiecinski C.J. (2000). Epoxide Hydrolases: Biochemistry and Molecular Biology. Chem. Biol. Interact..

[7] Morisseau C., Kodani S.D., Kamita S.G., Yang J., Lee K.S.S., Hammock B.D. (2021). Relative Importance of Soluble and Microsomal Epoxide Hydrolases for the Hydrolysis of Epoxy-Fatty Acids in Human Tissues. Int. J. Mol. Sci..

[8] Kodani S.D., Morisseau C. (2019). Role of Epoxy-Fatty Acids and Epoxide Hydrolases in the Pathology of Neuro-Inflammation. Biochimie.

[9] Gautheron J., Jéru I. (2020). The Multifaceted Role of Epoxide Hydrolases in Human Health and Disease. Int. J. Mol. Sci..

[10] Decker M., Adamska M., Cronin A., Di Giallonardo F., Burgener J., Marowsky A., Falck J.R., Morisseau C., Hammock B.D., Gruzdev A. (2012). EH3 (ABHD9): The First Member of a New Epoxide Hydrolase Family with High Activity for Fatty Acid Epoxides. J. Lipid Res..

[11] Cao L., Ba Y., Chen F., Li D., Zhang S., Zhang H. (2025). The Prognostic Significance of Epoxide Hydrolases in Colorectal Cancer. Biochem. Biophys. Rep..

[12] Shen N., Gao G., Lu X., Jin J., Lin L., Qian M., Qin Y. (2024). Comprehensive Analysis of the Immune Implication of EPHX4 Gene in Laryngeal Squamous Cell Carcinoma. Braz. J. Otorhinolaryngol..

[13] Tuppurainen H., Laurila N., Nätynki M., Eshraghi L., Tervasmäki A., Erichsen L., Sørensen C.S., Pylkäs K., Winqvist R., Peltoketo H. (2024). PALB2-Mutated Human Mammary Cells Display a Broad Spectrum of Morphological and Functional Abnormalities Induced by Increased TGFβ Signaling. Cell. Mol. Life Sci..

[14] Triepels R., Smeitink J., Loeffen J., Smeets R., Buskens C., Trijbels F., van den Heuvel L. (1999). The Human Nuclear-Encoded Acyl Carrier Subunit (NDUFAB1) of the Mitochondrial Complex I in Human Pathology. J. Inherit. Metab. Dis..

[15] Stein I.S., Gottfried A., Zimmermann J., von Mollard G.F. (2009). TVP23 Interacts Genetically with the Yeast SNARE VTI1 and Functions in Retrograde Transport from the Early Endosome to the Late Golgi. Biochem. J..

[16] Mino-Kenudson M., Schalper K., Cooper W., Dacic S., Hirsch F.R., Jain D., Lopez-Rios F., Tsao M.S., Yatabe Y., Beasley M.B. (2022). Predictive Biomarkers for Immunotherapy in Lung Cancer: Perspective From the International Association for the Study of Lung Cancer Pathology Committee. J. Thorac. Oncol..

[17] Gibney G.T., Weiner L.M., Atkins M.B. (2016). Predictive Biomarkers for Checkpoint Inhibitor-Based Immunotherapy. Lancet Oncol..

[18] Taranto D., Kloosterman D.J., Akkari L. (2024). Macrophages and T Cells in Metabolic Disorder-Associated Cancers. Nat. Rev. Cancer.

[19] Huang S., Shi J., Shen J., Fan X. (2025). Metabolic Reprogramming of Neutrophils in the Tumor Microenvironment: Emerging Therapeutic Targets. Cancer Lett..

[20] Li Y., Zheng Y., Huang J., Nie R.-C., Wu Q.-N., Zuo Z., Yuan S., Yu K., Liang C.-C., Pan Y.-Q. (2025). CAF-Macrophage Crosstalk in Tumour Microenvironments Governs the Response to Immune Checkpoint Blockade in Gastric Cancer Peritoneal Metastases. Gut.

[21] Bao L., Li Y., Hu X., Gong Y., Chen J., Huang P., Tan Z., Ge M., Pan Z. (2024). Targeting SIGLEC15 as an Emerging Immunotherapy for Anaplastic Thyroid Cancer. Int. Immunopharmacol..

[22] Asciolla J.J., Wu X., Adamopoulos C., Gavathiotis E., Poulikakos P.I. (2025). Resistance Mechanisms and Therapeutic Strategies of CDK4 and CDK6 Kinase Targeting in Cancer. Nat. Cancer.

[23] Chou J., Quigley D.A., Robinson T.M., Feng F.Y., Ashworth A. (2020). Transcription-Associated Cyclin-Dependent Kinases as Targets and Biomarkers for Cancer Therapy. Cancer Discov..

[24] Gharbi S.I., Pelletier L.A., Espada A., Gutiérrez J., Sanfeliciano S.M.G., Rauch C.T., Ganado M.P., Baquero C., Zapatero E., Zhang A. (2022). Crystal Structure of Active CDK4-Cyclin D and Mechanistic Basis for Abemaciclib Efficacy. npj Breast Cancer.

[25] Zhang D., Baldwin P., Leal A.S., Carapellucci S., Sridhar S., Liby K.T. (2019). A Nano-Liposome Formulation of the PARP Inhibitor Talazoparib Enhances Treatment Efficacy and Modulates Immune Cell Populations in Mammary Tumors of BRCA-Deficient Mice. Theranostics.

[26] Uxa S., Castillo-Binder P., Kohler R., Stangner K., Müller G.A., Engeland K. (2021). Ki-67 Gene Expression. Cell Death Differ..

[27] Teli G., Maji L., Pal R., Maheshwari N., Purawarga Matada G.S., Chawla P.A., Chawla V. (2025). Recent Advancements in Mechanistic Research, Therapeutic Potential, and Structure-Activity Relationships of Aurora Kinase Inhibitors in Cancer Therapies. Bioorganic Chem..

[28] Love M.I., Huber W., Anders S. (2014). Moderated Estimation of Fold Change and Dispersion for RNA-Seq Data with DESeq2. Genome Biol..

[29] Pontén F., Jirström K., Uhlen M. (2008). The Human Protein Atlas—A Tool for Pathology. J. Pathol..

[30] Bindea G., Mlecnik B., Tosolini M., Kirilovsky A., Waldner M., Obenauf A.C., Angell H., Fredriksen T., Lafontaine L., Berger A. (2013). Spatiotemporal Dynamics of Intratumoral Immune Cells Reveal the Immune Landscape in Human Cancer. Immunity.

[31] Hänzelmann S., Castelo R., Guinney J. (2013). GSVA: Gene Set Variation Analysis for Microarray and RNA-Seq Data. BMC Bioinform..

[32] Li T., Fu J., Zeng Z., Cohen D., Li J., Chen Q., Li B., Liu X.S. (2020). TIMER2.0 for Analysis of Tumor-Infiltrating Immune Cells. Nucleic Acids Res..

[33] Gao J., Aksoy B.A., Dogrusoz U., Dresdner G., Gross B., Sumer S.O., Sun Y., Jacobsen A., Sinha R., Larsson E. (2013). Integrative Analysis of Complex Cancer Genomics and Clinical Profiles Using the cBioPortal. Sci. Signal..

[34] Chandrashekar D.S., Karthikeyan S.K., Korla P.K., Patel H., Shovon A.R., Athar M., Netto G.J., Qin Z.S., Kumar S., Manne U. (2022). UALCAN: An Update to the Integrated Cancer Data Analysis Platform. Neoplasia.

[35] Wu T., Hu E., Xu S., Chen M., Guo P., Dai Z., Feng T., Zhou L., Tang W., Zhan L. (2021). clusterProfiler 4.0: A Universal Enrichment Tool for Interpreting Omics Data. Innovation.

[36] Szklarczyk D., Nastou K., Koutrouli M., Kirsch R., Mehryary F., Hachilif R., Hu D., Peluso M.E., Huang Q., Fang T. (2025). The STRING Database in 2025: Protein Networks with Directionality of Regulation. Nucleic Acids Res..

