# Supplementary material for: Integrative Analysis of EPHX4 as a Novel Prognostic and Diagnostic Biomarker in Lung Adenocarcinoma
Source: Int J Mol Sci. 2025 May 26;26(11):5095. doi: 10.3390/ijms26115095 (PMC12154108; doi:10.3390/ijms26115095)
Supplement: Supplementary file 1 [file ijms-26-05095-s001.zip › ijms-3649137-supplementary.pdf]

Figure S1. EPHX4 exhibited the best diagnostic value

ROC curve analysis of the six genes resulting from the intersection of differentially expressed genes and oxidative stress-related genes revealed that EPHX4 possessed the highest AUC.

Figure S2. Single-cell atlas of EPHX4 expression

Single-cell data from four distinct sample sources within the TISCH database collectively indicated predominant expression of EPHX4 in $CD8^+$ T cells, $CD8^+$ T exhausted cells, and malignant cells.

Figure S3. Prognostic factor analysis and nomogram model construction

(A, B, C) Forest plots showing the results of univariate and multivariate Cox regression analyses for OS/DSS/PFI of TCGA-LUAD patients. (D) Nomogram constructed based on univariate and multivariate Cox regression to estimate 1-year, 3-year, and 5-year survival probabilities (OS/DSS/PFI). (E) Calibration curves indicated a high accuracy between predicted values and actual observed values.

Figure S4. Immune infiltration analysis via TIMER2.0 database

Across various cancer types, EPHX4 expression generally exhibited a positive correlation with $CD4^+$ T cell, neutrophil, and macrophage infiltration, and a negative correlation with B cell infiltration.

Figure S5. Genetic variations and CpG sites' methylation of EPHX4

(A) Genetic variations of EPHX4 were observed in LUAD via the cBioPortal network. (B) The copy number variation (CNA) of *EPHX4* was positively correlated with mRNA expression. (C) The MethSurv database indicated that most of these CpG sites of *EPHX4* exhibited low methylation in LUAD.

Figure S6. Identification of DEGs and functional enrichment analysis

(A) Co-expression between *EPHX4* and cell cycle-related genes. (B) DEGs between high and low *EPHX4* expression groups. (C) 25 significantly upregulated or downregulated genes. (D) GO and KEGG enrichment analysis. (E) GO and KEGG terms corresponding to DEGs. (F) EMAP network diagram visually displaying the connections between functional modules, with line thickness representing pairwise similarity between terms; thicker lines indicate higher similarity, and thinner lines indicate lower similarity. GO: Gene Ontology; KEGG: Kyoto Encyclopedia of Genes and Genomes. ns: $p \geq 0.05$; *$p<0.05$; **$p<0.01$; ***$p<0.001$.

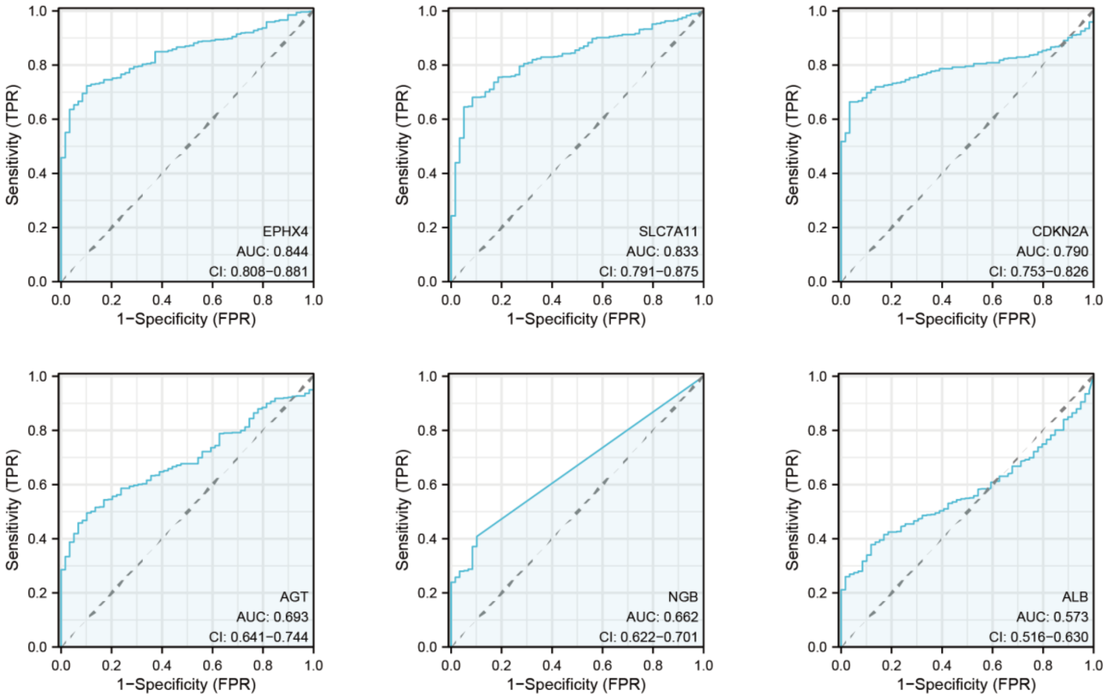

Figure S1. EPHX4 exhibited the best diagnostic value

ROC curve analysis of the six genes resulting from the intersection of differentially expressed genes and oxidative stress-related genes revealed that EPHX4 possessed the highest AUC.

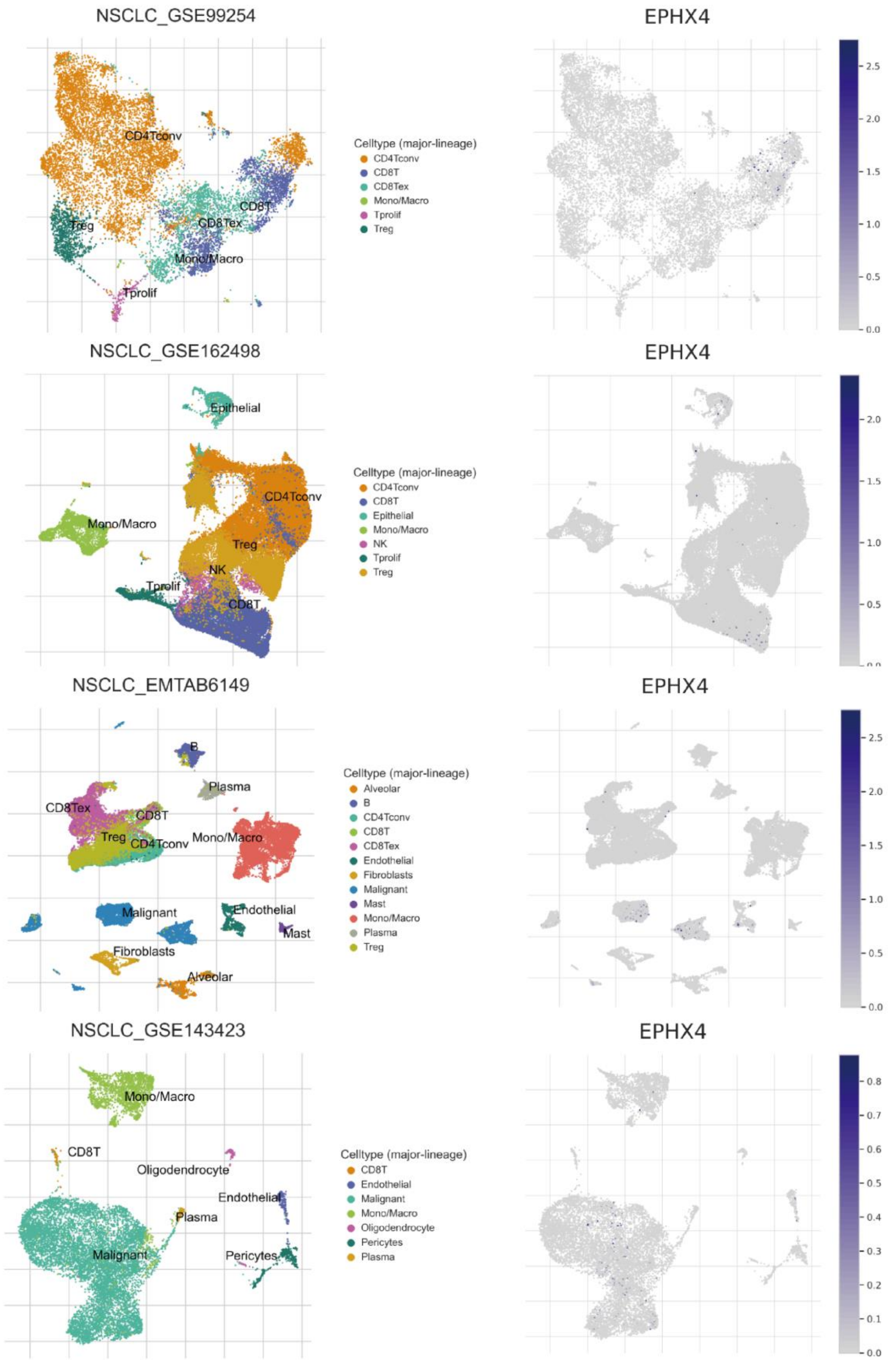

Figure S2. Single-cell atlas of EPHX4 expression Single-cell data from four distinct sample sources within the TISCH database collectively indicated predominant expression of EPHX4 in CD8+ T cells, CD8+ T exhausted cells, and malignant cells.

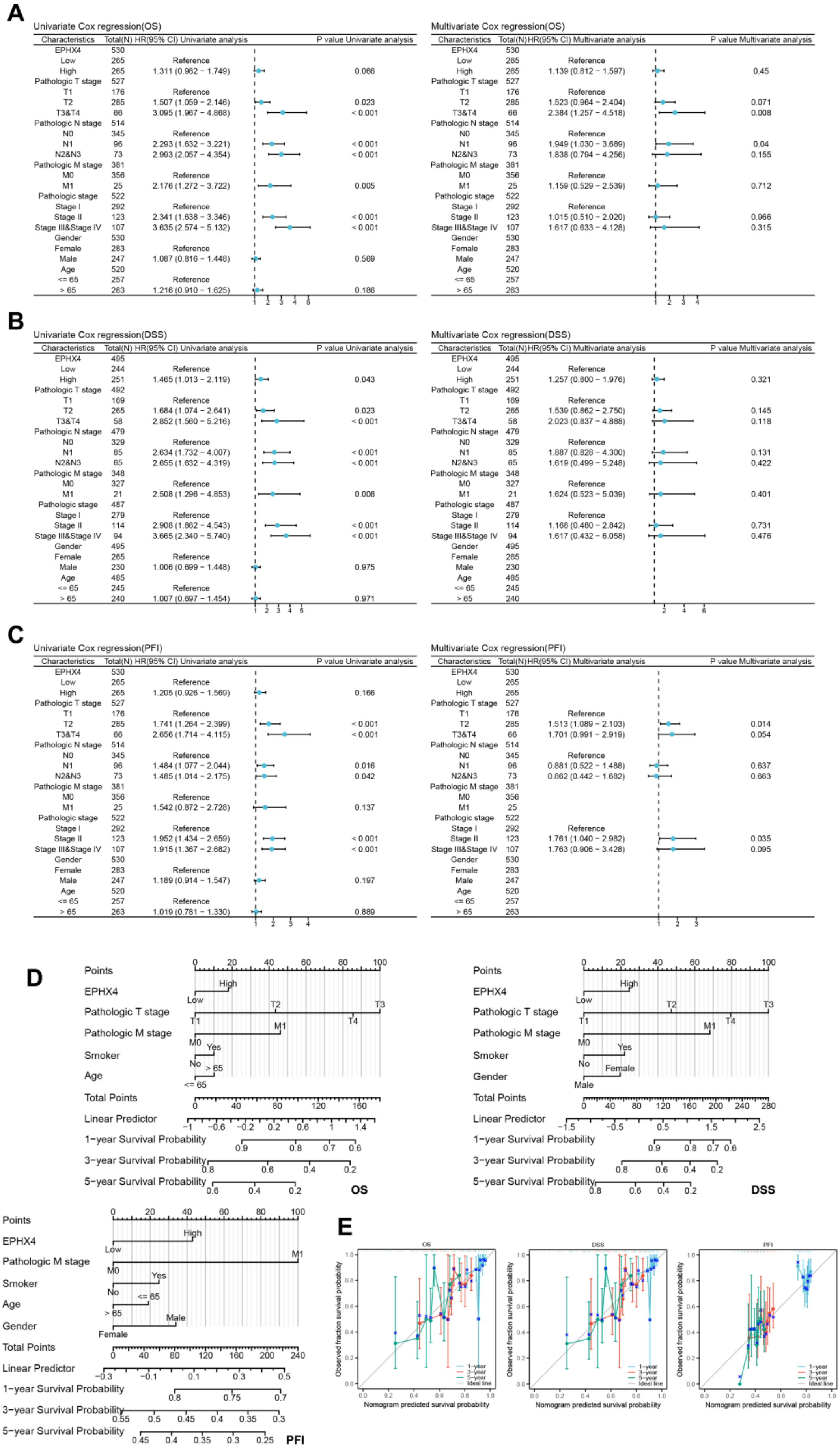
A
Univariate Cox regression(OS)
Characteristics Total(N) HR(95% CI) Univariate analysis P value Univariate analysis
EPHX4 530
Low 265 Reference
High 265 1.311 (0.982 − 1.749) 0.066
Pathologic T stage 527
T1 176 Reference
T2 285 1.507 (1.059 − 2.146) 0.023
T3&T4 66 3.095 (1.967 − 4.868) < 0.001
Pathologic N stage 514
N0 345 Reference
N1 96 2.293 (1.632 − 3.221) < 0.001
N2&N3 73 2.993 (2.057 − 4.354) < 0.001
Pathologic M stage 381
M0 356 Reference
M1 25 2.176 (1.272 − 3.722) 0.005
Pathologic stage 522
Stage I 292 Reference
Stage II 123 2.341 (1.638 − 3.346) < 0.001
Stage III&Stage IV 107 3.635 (2.574 − 5.132) < 0.001
Gender 530
Female 283 Reference
Male 247 1.087 (0.816 − 1.448) 0.569
Age 520
<= 65 257 Reference
> 65 263 1.216 (0.910 − 1.625) 0.186
Multivariate Cox regression(OS)
Characteristics Total(N) HR(95% CI) Multivariate analysis P value Multivariate analysis
EPHX4 530
Low 265 Reference
High 265 1.139 (0.812 − 1.597) 0.45
Pathologic T stage 527
T1 176 Reference
T2 285 1.523 (0.964 − 2.404) 0.071
T3&T4 66 2.384 (1.257 − 4.518) 0.008
Pathologic N stage 514
N0 345 Reference
N1 96 1.949 (1.030 − 3.689) 0.04
N2&N3 73 1.838 (0.794 − 4.256) 0.155
Pathologic M stage 381
M0 356 Reference
M1 25 1.159 (0.529 − 2.539) 0.712
Pathologic stage 522
Stage I 292 Reference
Stage II 123 1.015 (0.510 − 2.020) 0.966
Stage III&Stage IV 107 1.617 (0.633 − 4.128) 0.315
Gender 530
Female 283
Male 247
Age 520
<= 65 257
> 65 263
B
Univariate Cox regression(DSS)
Characteristics Total(N) HR(95% CI) Univariate analysis P value Univariate analysis
EPHX4 495
Low 244 Reference
High 251 1.465 (1.013 − 2.119) 0.043
Pathologic T stage 492
T1 169 Reference
T2 265 1.684 (1.074 − 2.641) 0.023
T3&T4 58 2.852 (1.560 − 5.216) < 0.001
Pathologic N stage 479
N0 329 Reference
N1 85 2.634 (1.732 − 4.007) < 0.001
N2&N3 65 2.655 (1.632 − 4.319) < 0.001
Pathologic M stage 348
M0 327 Reference
M1 21 2.508 (1.296 − 4.853) 0.006
Pathologic stage 487
Stage I 279 Reference
Stage II 114 2.908 (1.862 − 4.543) < 0.001
Stage III&Stage IV 94 3.665 (2.340 − 5.740) < 0.001
Gender 495
Female 265 Reference
Male 230 1.006 (0.699 − 1.448) 0.975
Age 485
<= 65 245 Reference
> 65 240 1.007 (0.697 − 1.454) 0.971
Multivariate Cox regression(DSS)
Characteristics Total(N) HR(95% CI) Multivariate analysis P value Multivariate analysis
EPHX4 495
Low 244 Reference
High 251 1.257 (0.800 − 1.976) 0.321
Pathologic T stage 492
T1 169 Reference
T2 265 1.539 (0.862 − 2.750) 0.145
T3&T4 58 2.023 (0.837 − 4.888) 0.118
Pathologic N stage 479
N0 329 Reference
N1 85 1.887 (0.828 − 4.300) 0.131
N2&N3 65 1.619 (0.499 − 5.248) 0.422
Pathologic M stage 348
M0 327 Reference
M1 21 1.624 (0.523 − 5.039) 0.401
Pathologic stage 487
Stage I 279 Reference
Stage II 114 1.168 (0.480 − 2.842) 0.731
Stage III&Stage IV 94 1.617 (0.432 − 6.058) 0.476
Gender 495
Female 265
Male 230
Age 485
<= 65 245
> 65 240
C
Univariate Cox regression(PFI)
Characteristics Total(N) HR(95% CI) Univariate analysis P value Univariate analysis
EPHX4 530
Low 265 Reference
High 265 1.205 (0.926 − 1.569) 0.166
Pathologic T stage 527
T1 176 Reference
T2 285 1.741 (1.264 − 2.399) < 0.001
T3&T4 66 2.656 (1.714 − 4.115) < 0.001
Pathologic N stage 514
N0 345 Reference
N1 96 1.484 (1.077 − 2.044) 0.016
N2&N3 73 1.485 (1.014 − 2.175) 0.042
Pathologic M stage 381
M0 356 Reference
M1 25 1.542 (0.872 − 2.728) 0.137
Pathologic stage 522
Stage I 292 Reference
Stage II 123 1.952 (1.434 − 2.659) < 0.001
Stage III&Stage IV 107 1.915 (1.367 − 2.682) < 0.001
Gender 530
Female 283 Reference
Male 247 1.189 (0.914 − 1.547) 0.197
Age 520
<= 65 257 Reference
> 65 263 1.019 (0.781 − 1.330) 0.889
Multivariate Cox regression(PFI)
Characteristics Total(N) HR(95% CI) Multivariate analysis P value Multivariate analysis
EPHX4 530
Low 265
High 265
Pathologic T stage 527
T1 176 Reference
T2 285 1.513 (1.089 − 2.103) 0.014
T3&T4 66 1.701 (0.991 − 2.919) 0.054
Pathologic N stage 514
N0 345 Reference
N1 96 0.881 (0.522 − 1.488) 0.637
N2&N3 73 0.862 (0.442 − 1.682) 0.663
Pathologic M stage 381
M0 356
M1 25
Pathologic stage 522
Stage I 292 Reference
Stage II 123 1.761 (1.040 − 2.982) 0.035
Stage III&Stage IV 107 1.763 (0.906 − 3.428) 0.095
Gender 530
Female 283
Male 247
Age 520
<= 65 257
> 65 263
D
Points
EPHX4
Pathologic T stage
Pathologic M stage
Smoker
Age
Total Points
Linear Predictor
1−year Survival Probability
3−year Survival Probability
5−year Survival Probability
OS
Points
EPHX4
Pathologic T stage
Pathologic M stage
Smoker
Gender
Total Points
Linear Predictor
1−year Survival Probability
3−year Survival Probability
5−year Survival Probability
DSS
Points
EPHX4
Pathologic M stage
Smoker
Age
Gender
Total Points
Linear Predictor
1−year Survival Probability
3−year Survival Probability
5−year Survival Probability
PFI
E
OS
DSS
PFI
Observed fraction survival probability
Nomogram predicted survival probability
1−year
3−year
5−year
Ideal line

Figure S3. Prognostic factor analysis and nomogram model construction

(A, B, C) Forest plots showing the results of univariate and multivariate Cox regression analyses for OS/DSS/PFI of TCGA-LUAD patients. (D) Nomogram constructed based on univariate and multivariate Cox regression to estimate 1-year, 3-year, and 5-year survival probabilities (OS/DSS/PFI). (E) Calibration curves indicated a high accuracy between predicted values and actual observed values.

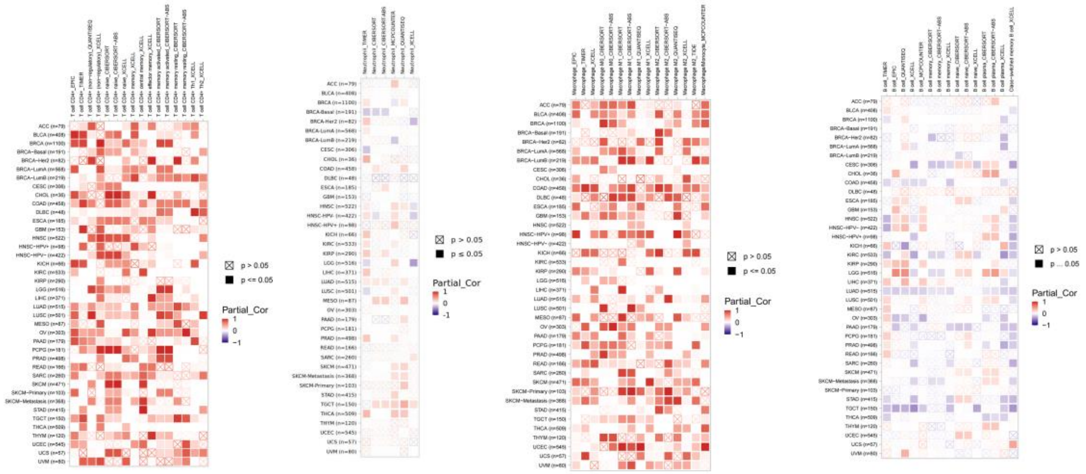

Figure S4. Immune infiltration analysis via TIMER2.0 database

Across various cancer types, EPHX4 expression generally exhibited a positive correlation with $CD4^{+}$ T cell, neutrophil, and macrophage infiltration, and a negative correlation with B cell infiltration.

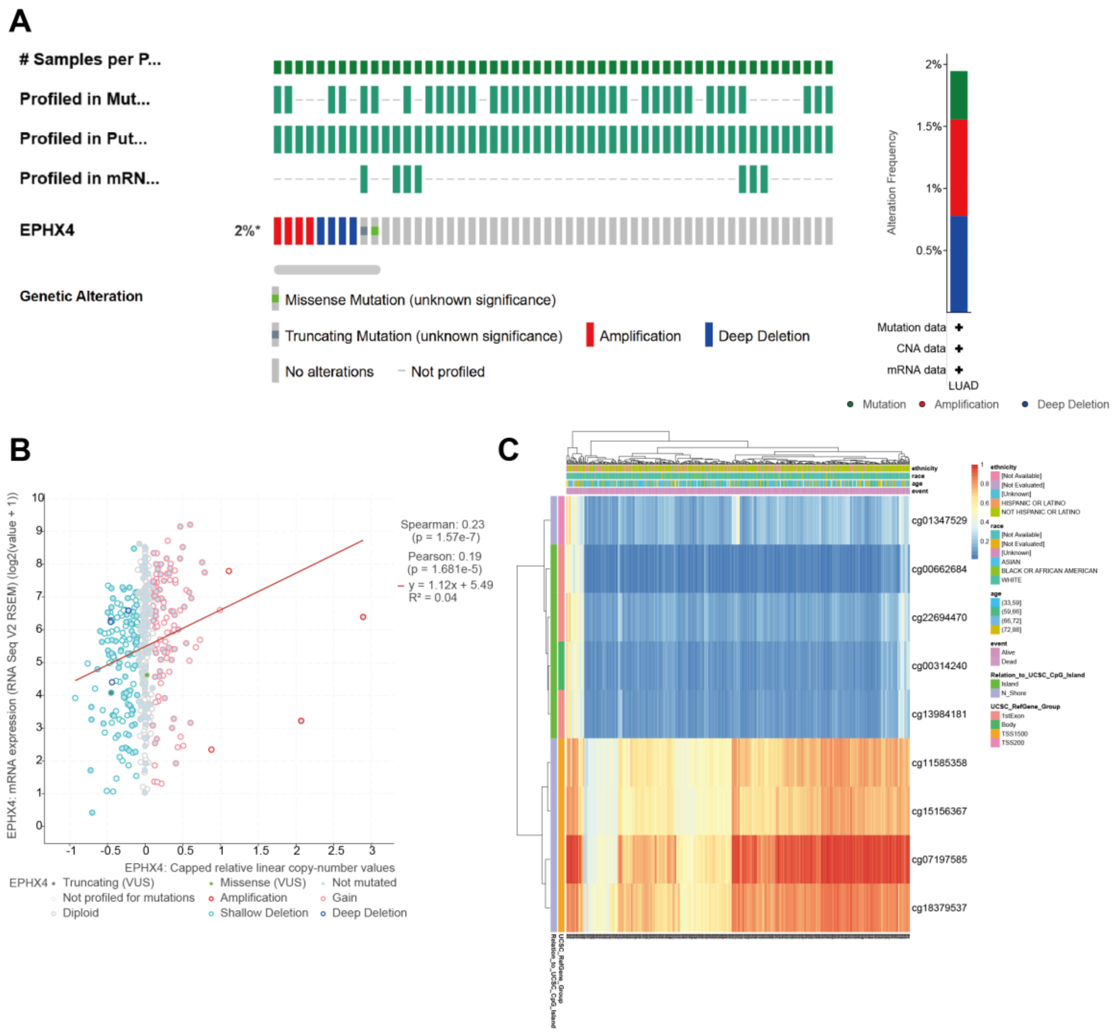

Figure S5. Genetic variations and CpG sites' methylation of EPHX4

(A) Genetic variations of EPHX4 were observed in LUAD via the cBioPortal network. (B) The copy number variation (CNA) of *EPHX4* was positively correlated with mRNA expression. (C) The MethSurv database indicated that most of these CpG sites of *EPHX4* exhibited low methylation in LUAD.

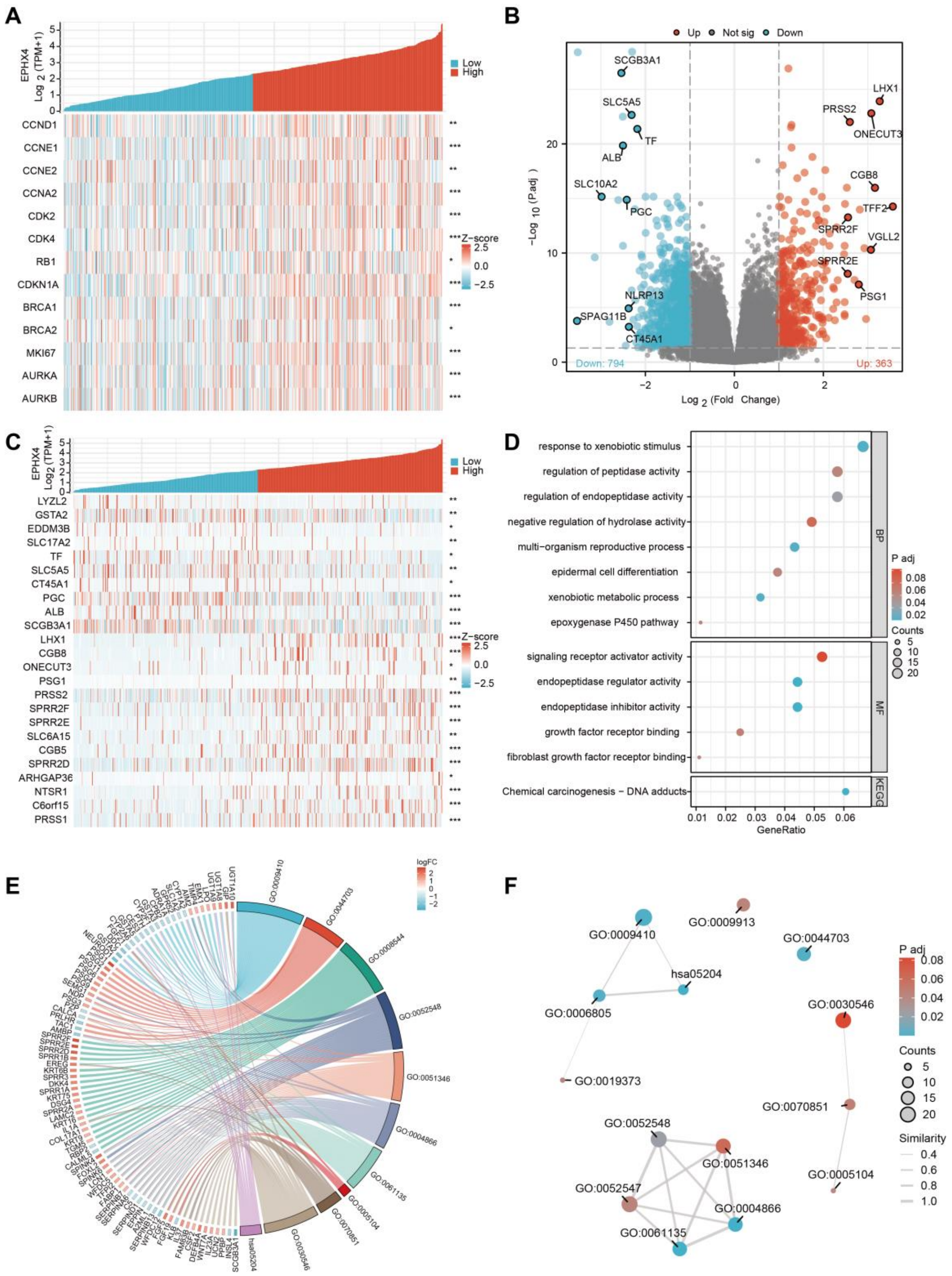

Figure S6. Identification of DEGs and functional enrichment analysis

(A) Co-expression between *EPHX4* and cell cycle-related genes. (B) DEGs between high and low *EPHX4* expression groups. (C) 25 significantly upregulated or downregulated genes. (D) GO and KEGG enrichment analysis. (E) GO and KEGG terms corresponding to DEGs. (F) EMAP network diagram visually displaying the connections between functional modules, with line thickness representing pairwise similarity between terms; thicker lines indicate higher similarity, and thinner lines indicate lower similarity. GO: Gene Ontology; KEGG: Kyoto Encyclopedia of Genes and Genomes. *p<0.05; **p<0.01; ***p<0.001.
